# The genetic backbone of ankylosing spondylitis: how knowledge of genetic susceptibility informs our understanding and management of disease

**DOI:** 10.1007/s00296-022-05174-5

**Published:** 2022-08-08

**Authors:** Marcus Kenyon, Sinead Maguire, Anna Rueda Pujol, Finbar O’Shea, Ross McManus

**Affiliations:** 1 Department of Clinical Medicine, Trinity Translational Medicine Institute, Trinity College Dublin, Dublin, Ireland; 2 Department of Rheumatology, St James’ Hospital, Dublin, Ireland

**Keywords:** Ankylosing spondylitis, Spondyloarthropathy, Genetics, Therapeutics, Polygenic risk, Comorbidity

## Abstract

Ankylosing spondylitis (AS) is a seronegative, chronic inflammatory arthritis with high genetic burden. A strong association with HLA-B27 has long been established, but to date its contribution to disease aetiology remains unresolved. Recent insights through genome wide studies reveal an increasing array of immunogenetic risk variants extraneous to the HLA complex in AS cohorts. These genetic traits build a complex profile of disease causality, highlighting several molecular pathways associated with the condition. This and other evidence strongly implicates *T*-cell-driven pathology, revolving around the *T* helper 17 cell subset as an important contributor to disease. This prominence of the *T* helper 17 cell subset has presented the opportunity for therapeutic intervention through inhibition of interleukins 17 and 23 which drive *T* helper 17 activity. While targeting of interleukin 17 has proven effective, this success has not been replicated with interleukin 23 inhibition in AS patients. Evidence points to significant genetic diversity between AS patients which may, in part, explain the observed refractoriness among a proportion of patients. In this review we discuss the impact of genetics on our understanding of AS and its relationship with closely linked pathologies. We further explore how genetics can be used in the development of therapeutics and as a tool to assist in the diagnosis and management of patients. This evidence indicates that genetic profiling should play a role in the clinician’s choice of therapy as part of a precision medicine strategy towards disease management.

## Introduction

Ankylosing spondylitis (AS) is a complex autoimmune disorder, presenting primarily as inflammation and tissue remodelling within the axial skeleton. The disease is highly heritable, with concordance rates of approximately 50% in monozygotic twins [1, 2]. First- and second-degree relatives are affected at rates of 8.2 and 1%, respectively [1]. Historically, much research focus was placed on the major histocompatibility complex (MHC) class I human leukocyte antigen (HLA) variant, HLA-B27, which is observed in approximately 90% of Caucasian AS patients [3]. Despite this intensive research, almost 50 years after its discovery, the mechanisms by which HLA-B27 contributes to pathogenicity remain largely unresolved. However, through the discovery of many genetic associations outside of the HLA complex over the past decade, it has become increasingly clear that HLA-B27 is not the sole genetic determinant of the disease, with many variants across the genome now known to contribute to AS susceptibility [4, 5]. Focusing on the non-HLA associations, most occur within or near immunologically relevant genes, while others are found in intergenic genomic regions which may have gene regulatory roles yet to be functionally characterised [5]. This diverse collection of disease associated genes illustrates the complex, polygenic nature of AS.

Over the past decade, many papers have discussed the genetic findings in AS, but few address how genetic findings can be evaluated and used to inform clinical decision making. Here, we discuss how genetics offers insight into the biomolecular pathways of AS pathology and its most closely related comorbidities, for some of which therapeutic strategies are available. We further examine the potential roles for genetics beyond HLA-B27 in the diagnosis and management of patients; examine the potential roles for genetics beyond HLA-B27 in the diagnosis and management of patients.

## Search strategy

This article employed a modified form of narrative review in accordance with recent recommendations on composing a biomedical narrative review [6]. Searches were performed using the MEDLINE (PubMed) and SCOPUS databases, and Google Scholar search engine for the following criteria:

((“Ankylosing spondylitis” OR “axial spondyloarthropathy”) AND (“GWAS” OR “Polygene Risk” OR “DMARD”)) ((“Ankylosing spondylitis” OR “axial spondyloarthropathy”) AND (“Psoriasis” OR “Ulcerative colitis” OR “Crohn’s” OR “inflammatory bowel disease” OR “uveitis”) AND (“genetics” OR “GWAS”)). Search was confined between dates of January 2008 to January 2022. Original research and review articles focussing on the relationship between genetics and phenotype were evaluated.

Due to a significant body of work concerning serum cytokines, HLA-B27 and classification of disease which pre-dates the era of genome wide association, separate searches were performed between dates of earliest database record to January 2022. Original research and review articles concerning serum or tissue cytokines, or the role of HLA-B27 in pathology were considered. Search terms used were ((“ankylosing spondylitis” OR “axial spondyloarthropathy”) AND (“cytokine” OR “signalling”)); ((“ankylosing spondylitis” OR “axial spondyloarthropathy”) AND (“HLA-B27”) AND (“mechanism”)). Information on disease classification and diagnostic criteria from advisory meetings was derived from the authors’ prior knowledge.

## Genetic susceptibility to AS: antigen presentation plays a central but poorly understood role

Contemporary understanding of genetic susceptibility to complex diseases such as AS has been shaped primarily through genome wide association studies (GWAS) which have allowed the identification of genome regions associated with these diseases. These studies rely on the genotyping of up to a million genetic variants distributed across the genome, utilising large case and control sample sizes to differentiate genomic regions (loci) associated with the disease phenotype. GWAS focus almost exclusively on common variants (occurring in > 1% of the population) which involve a single nucleotide base change, referred to as single nucleotide polymorphisms or variants (SNP or SNV). The largest GWAS study performed to date on AS was in 2013 by the International Genetics of Ankylosing Spondylitis (IGAS) consortium [5] which identified 25 associated loci using the Illumina Immunochip. A succession of other studies of AS and related conditions has now identified 115 independently associated SNPs in over 90 genomic regions [7]. Many of these variants are located near genes of immunological interest. The majority of variants identified are not within the protein coding sections of genes, and are considered most likely to affect the regulation of gene expression [8]. Exceptions to this include variants within the endoplasmic reticulum amidopeptidase genes, *ERAP1* and *ERAP2*, which result in changes to the amino acid sequence which are believed to alter protein function [5].

ERAP1 and ERAP2 are enzymes responsible for the trimming of peptide antigens to an appropriate size prior to loading onto MHC class I molecules, including HLA-B27 [9]. Thus they play a key role in the selection of endogenous antigens for presentation to *T*-cells. *ERAP1* variants show strong interaction (epistasis) with *HLA-B27* in genetic analyses [10–12]. Variants of *ERAP1* which reduce the enzyme’s activity are protective against AS [13]. This suggests that efficient loading of antigens onto HLA-B27 is a driving mechanism of AS, which is in line with what is known as the *arthritogenic peptide* model of disease. This model is similar to other autoantigen theories of autoimmunity and posits that autoimmunity results from the presentation of self-peptides which elicit an inappropriate response from *T* cells [14]. These *T* cells then mount a concerted immunological attack on a normal host protein which becomes a self-target, causing the inflammation and tissue destruction associated with autoimmune disease. HLA-B27 is known to be unusual in its antigen binding properties which could play a key role in facilitating this type of response. It has a restricted library of antigens to which it can bind, which may favour inappropriate antigen presentation as a driver of HLA-B27 associated disease [15]. However, there remains some evidence which appears incongruous to the arthritogenic peptide hypothesis. For example, ERAP2, which is independently associated to AS and performs a role similar to ERAP1, does not appear to have an epistatic interaction with HLA-B27 [12, 16, 17]. Furthermore, the genetic association of *ERAP1* to AS has not been demonstrated in the Han Chinese population, despite a high prevalence of HLA-B27 [18]. It has previously been shown that peptides bound to HLA-B27 may modulate interactions with killer immunoglobulin-like receptors (KIR) [19]. A recent study has demonstrated increased AS risk linked to interactions between specific combinations of KIR subclass and HLA subtype molecules [20]. However, the researchers found no preferential KIR subtype favouring ERAP1 in B27 + AS patients, suggesting that the epistatic interaction of ERAP1 and HLA-B27 does not influence the selection of KIR subtypes [20]. Finally, while several studies have attempted to implicate auto-reactive or arthritogenic peptides, none have yet been identified [21]. Other possible mechanisms for HLA-B27’s pathological involvement have been suggested, all of which centre on irregularities stemming from its unusual molecular structure. One such mechanism proposes that nascent heavy chains of HLA-B27 molecules dimerise via disulphide bonds within the endoplasmic reticulum (ER) [22] and accumulate, provoking the unfolded protein response. This is a stress response mechanism within the ER, and results in the upregulation of inflammatory cytokine signalling, including TNF-α, IL-23 and IL-6 [23], all of which show increased concentrations in the serum of AS patients [24, 25]. Irrespective of the mechanism by which HLA-B27 causes disease, a significant body of genetic evidence supports altered immune cell function, with a particularly prominent role for dysregulated cytokine signalling, as a pathological mechanism in AS.

## Non-HLA genetics points to altered lymphocyte function

**Fig. 1:**
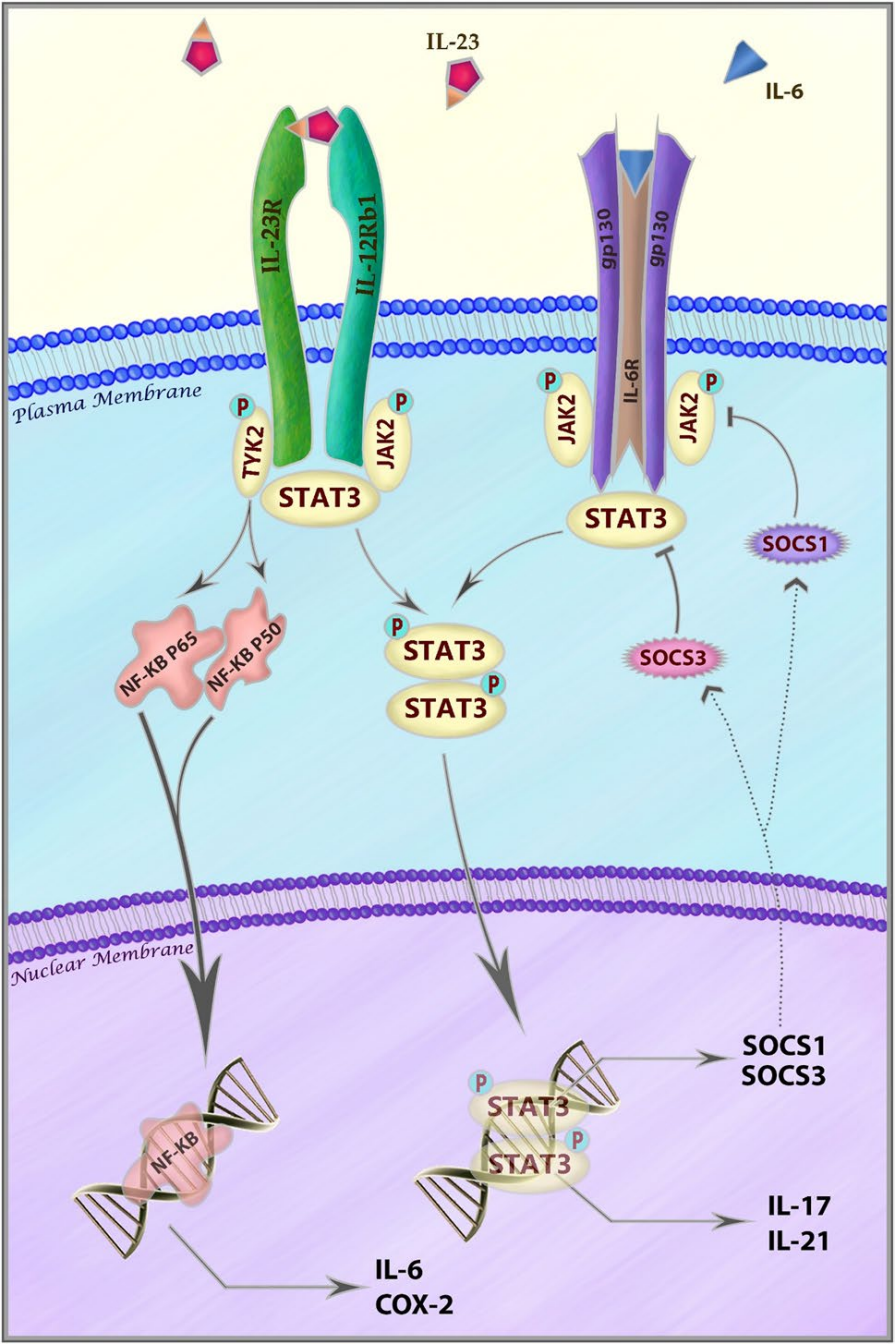
The central role of JAK/STAT signalling pathway in IL-17 producing *T*-cells. IL-6 and IL-23 signals are transduced via JAK2 and TYK2 to STAT3 which triggers production of the effector molecules IL-17 and IL-21. Furthermore, activation of NF-κB by IL-23 results in production of IL-6, further amplifying IL-17 and IL-21 production. Negative feedback is provided by increased expression of SOCS1 and SOCS3 which acto to inhibit JAK2 and STAT3, respectively, limiting further autocrine and paracrine response [31–34]

Beyond the HLA complex on chromosome 6, genetic evidence strongly points to lymphocytes and, in particular, *T*-cell function as important players in AS. Variants affecting genes of molecular pathways involved in Th17 cell differentiation and function, including *IL23*, *IL12B* (a subunit common to both IL-12 and IL-23 cytokines), and *IL6* have all been identified through GWAS of AS (Table 1) [5, 26]. It should be noted that the *IL23R* coding variant, rs11209026 (Arg381Gln), which shows reduced IL-23R signalling, actually plays a protective role in AS and other inflammatory conditions, including psoriasis and inflammatory bowel disease (IBD), indicating that attenuation of IL-23 signalling is protective against these conditions [5, 27–29]. IL-23 and IL-6 are key components of the IL-23/IL-17 cell signalling axis which is responsible for the differentiation and function of T-helper 17 (Th17) cells [26]. Th17 cells are of significant interest, since they are thought to drive a number of diseases of auto-immune aetiology, including multiple sclerosis, rheumatoid arthritis, psoriasis, Crohn’s disease and ulcerative colitis [26]. Evidence is growing to suggest these cells also play a prominent role in coordinating AS pathology [30]. Together with cytokine and receptor variants, further associations have been identified near their downstream signalling molecules, including the *JAK2* and *TYK2* genes, which together play a critical role in the IL-6 and IL-23 signal transduction pathways (among others) (Fig. 1) [31–34]. JAK2 and TYK2 function through the Signal Transduction and Activator of Transcription proteins, including prominently STAT3, leading to increased expression of IL-17A and IL-21, important effectors of Th17 cells (Fig. 1) [7, 35]. Given these associations with AS at the genomic level, it is interesting that the IL-23/IL-17 axis has previously been implicated in bone restructuring through inhibition of osteoclastogenesis or induction of osteoblastogenesis [36, 37]. As discussed below, this pathway continues to be heavily investigated from the perspective of therapeutic interventions.

**Table 1:** A brief overview of most strongly implicated genes in AS and their known functions [5, 38–41]

Gene	Known function(s)
HLA-B27	Antigen presentation to T-lymphocytes
ERAP1	Preparation of peptides by trimming prior to MHC class I binding
ERAP2	Preparation of peptides by trimming prior to MHC class I binding
IL-23R	IL-23 signal transduction in *T* cells and NK cells
RUNX3	T-helper I (Th1) cell differentiation. Downregulation of Th2 cell activity
TYK2	Cytokine signal transduction via the JAK/STAT pathway
PTGER4	*T* cell function, cyclooxygenase 2 regulation
IL12B	Subunit for IL12 and IL23 heterodimeric cytokine signalling molecules
BACH2	Transcriptional regulator with role in lymphocyte differentiation/function
CARD9	Adaptor protein involved in TLR and NOD2 pathways
IL6R	Receptor for IL-6 cytokine
TNFRSF1A	Receptor for TNFSF2/TNF-alpha
FCGR2A	Phagocytic receptor for IgG
UBE2E3	Ubiquitin tagging of proteins for degradation
ZMIZ1	Regulator of transcription factor activity, including SMAD3/4
NKX2-3	Transcription factor with possible role in cellular differentiation
SH2B3	Cytokine signalling regulator. JAK/STAT signalling of EPO transduction
GPR65	Glycosphingolipid receptor with role in microbial phagocytosis and lysosomal pH
NOS2	Production of nitric oxide. Downstream promotion of IL-6 and IL-8
ICOSLG	T-cell proliferation. *B*-cell proliferation and differentiation to plasma cells
TNP2	No known immunological role
DNMT3B	DNA methylation
IL2RA	Controlling of *T* regulatory cell activity
IL27	Lymphocyte proliferation and Th1 cell differentiation
GPR35	Receptor with a role in metabolism of tryptophan
INAVA	Pathogen Recognition Receptor Function and cytokine secretion

## Genetic commonality with extra-articular manifestations

AS is closely related to a number of other seronegative autoimmune diseases. These diseases are frequently found in the setting of AS, sharing substantial genetic commonality and are considered extra articular manifestations (EAMs) of AS from a clinical perspective. The most common of these are acute anterior uveitis (AAU); IBD, including both Crohn’s disease (CD) and ulcerative colitis (UC); and psoriasis [42].

For each of these conditions, a genetic profile of disease-associated variants has been identified through GWAS and some of these variants are found across multiple EAMs. For example, pleiotropy in *HLA-B27*, *ERAP1/2* and multiple genes of the IL-23/IL-17 pathway, among others, are observed across the disease group [38, 43]. Even though some variants are shared between these conditions, each condition has its own unique genetic fingerprint. The directionality or relative risk of shared variants may, thus differ between EAM’s and AS. This makes interpreting of the role of such shared variants in EAM’s complex [12].

AAU is the most frequently observed EAM, presenting in around 30–40% of AS patients at least once during their lifetime [44, 45]. HLA-B27 is the most prominent genetic association in AAU, observed in almost half of isolated cases. HLA-B27 positive AAU has a similar demographic profile to AS, chiefly affecting younger persons with a distinct male preponderance (approximately 2:1) [46]. Onset is typically acute, unilateral, and often recurring. Unsurprisingly, given the strong HLA-B27 link to AS, HLA-B27 positive AAU patients are twice as likely to develop AS as those without HLA-B27 [46, 47]. By contrast, HLA-B27 negative AAU is generally chronic, bilateral, and affects middle aged individuals with no perceivable gender bias [47, 48]. The first large scale GWAS studying AAU [43] confirmed the HLA-B27 association, and found common genetic associations in the *ERAP1*, *IL23R*, *IL12B* and *IL6R* genes, suggesting a shared AS/AAU aetiology involving antigen processing/presentation and the IL-23/IL-17 axis. Gene ‘deserts’ (extensive intergenic regions) in chromosomal locations of *2p15* and *21q22* are also linked to both conditions [43, 44]. The functions of these regions is not established, although alteration of cell specific regulatory elements, for example enhancers, in an obvious possibility. The NOS2 gene, encoding a synthase for nitric oxide which is a signalling molecule and inflammatory mediator with broad effects was also implicated. Through cross comparison to AS GWAS, several further common genetic trends were identified, with these variants showing the same directionality of risk in AS and AAU (variants can promote disease or protect against it). Importantly, associations unique to AAU were also found when AS + /AAU + and AS + /AAU- individuals were compared. These included *MERTK*, which contributes to anti-inflammatory IL-37 signalling and has been shown to promote resolution of inflammation, primarily through activity on macrophages/myeloid cells [49, 50]. MERTK has also been linked to several retinal disorders, including retinitis pigmentosa [39, 51]. Interestingly, there has been the suggestion of IL-37 signalling involvement in AS, though the *MERTK* gene specifically has not, as yet, been specifically linked to the disease [52]. Other unique AAU genetic risk alleles were linked to intergenic regions, again with no currently identified mechanism of action.

Of the remaining most common EAM’s, psoriasis is present in around 10–25% of AS patients, while UC and CD are collectively observed in under 10% of patients, though subclinical gut inflammation has been described in 50% of cases [53, 54]. In contrast to AAU, these EAMs have been studied extensively at a genetic level through multiple GWAS. A remarkable degree of genetic concordance is observed between AS, IBD and psoriasis, coinciding with this phenotypic overlap [38]. AS is found to be around 50% genetically correlated with each of UC, psoriasis and CD, with a total of 31 non-MHC loci shared between these diseases identified, of which 12 are strongly linked to all three conditions [38]. Of these 12, four (*IL23R*, *IL27*, *IL-2RA* and *SH2B3)* are linked to T-lymphocyte function via the IL-23/IL-17 axis [55], feeding into the JAK/STAT signalling pathway, which as discussed above, is a particularly prominent pathway in AS. Other shared genetic variants are related to genes with a variety of cellular functions, including DNA methylation and chromatin remodelling, protein degradation and cell receptors.

An interesting association common to inflammatory bowel disease and AS is the Familial Mediterranean Fever gene, MEFV. MEFV is associated to both UC and CD, and is also linked to increased risk of extra-intestinal manifestations [56]. A recent meta-analysis of Turkish and Iranian GWAS [57] found strong association of MEFV in AS patients, particularly in B27 negative cases, which may point to a compensatory role of MEFV in the absence of B27. The MEFV gene product, pyrin, has a variety of important functions potentially relevant to inflammatory disease causation, including in innate immunity, inflammatory responses (particularly to the interferon gamma pathway) and autophagy [58, 59].

A functional summary of the top 100 non-HLA AS associated genes currently listed on Open Targets database of genetic associations with disease show 95 are also common to psoriasis, UC, and Crohn’s (Table 2) [31, 42]. Lymphocyte function is most highly represented with a total of 19 linked genes, with *T*-cell function predominance [55]. Ten of these genes are involved in IL-23/IL-17 cytokine signalling or the downstream JAK/STAT signal transduction pathway [55]. Transcriptional regulation is also a highly prominent mechanism. Notably, only 5 genes directly linked to antigen processing and presentation are common to all of these diseases, these being *HLA-B27*, *ERAP1*, *CD226*, *LNPEP* and *PTPN22* [55]. A number of implicated genes involve phagocytosis and pathogen processing. These include a coding variant in the IgG Fc receptor gene, *FCGR2A*, and non-coding variants close to *GPR65*, *CARD9* and *INAVA* [5, 38, 39]. Each of these molecules performs a niche immunological role (Table 1). Other associations across the disease group include the nitric oxide synthase (*NOS2*) gene. A few genes are also associated to the TGF-β superfamily, a system critical to immune cell maturation, T-regulatory cell function and remodelling of tissue, including bone [60]. A total of 21 plausibly linked genes have immunologically obscure or unknown function.

**Table 2:** Functional summary of 95 most prominent shared susceptibility genes

Gene function	Gene count
Lymphocyte function (all)	19
* T*-cell	15^a^
* B*-cell	2^a^
Involved in IL-23/IL-17 or JAK/STAT pathway	10^a^
Transcriptional regulation	16
Antigen processing/presentation	5
Metabolism	5
Apoptosis	4
Protein degradation	4
Innate immune cell function	4
Tissue remodelling/TGF-β pathway	3
Cellular proliferation/differentiation	2
Cellular adhesion	2
Other/unknown function	21

^a^Some genes involved in multiple lymphocyte cell subsets/functions

## Can genetic testing improve the diagnosis of AS?

The diagnosis of AS is often challenging. One of the reasons for this is the lack of validated biomarkers available to assist clinicians. Diagnosis must, therefore, be informed by clinical examination and disease progression. The 1984 Modified New York Criteria remains the gold standard for diagnosis of AS, but formal diagnosis using these criteria is delayed until radiographic sacroiliitis—a feature of advanced disease—has presented [61]. More recent classification criteria proposed by ASAS in 2009 recognised non-radiographic axial spondyloarthropathy (nr-axSpA), which is regarded as an earlier form of AS (referred to as r-axSpA in the ASAS criteria) with inflammatory sacroiliitis detected on MRI but not on plain radiographs of the sacroiliac joints. Around 18% of patients diagnosed with nr-axSpA will progress to AS per decade; however, not all nr-axSpA will eventually progress to AS. In fact, some may later be diagnosed with other spondyloarthropathies [62–64]. This difficulty in successfully differentiating AS at an early stage contributes to significant delay in the diagnosis and commencement of treatment [65].

One possible solution to identify patients most at risk of progression to AS is to employ genetic biomarkers. Multiple studies demonstrate significant divergence between axSpA and AS cohorts both in phenotype and genotype [62, 66, 67]. Since no single genetic marker confers total genetic risk, one approach gaining increasing interest is to use aggregate measures of all known genetic risk factors. Polygenic risk scores (PRS) are one such composite measure, which involve the testing of all known susceptibility variants to derive a personalised risk score for the condition being evaluated. Scores are calculated from the sum of all risk alleles weighted by their individual effect sizes (odds ratios), as estimated by GWAS, to give a final, personalised, genomic risk score [68]. This type of approach can be applied to any complex disease phenotype and has demonstrated positive predictive value in such diseases as myocardial infarction, Alzheimer’s disease and childhood obesity [69–71]. An early study in AS by Li et al. demonstrated improved predictive value of PRS against HLA-B27 typing alone [72]. PRS have also demonstrated positive predictive value for disease severity in systemic lupus erythematosis [73]. These promising findings suggest that PRS could lead to improved capacity to discriminate early stage disease patients for whom increased clinical monitoring or different therapeutic approaches may be of benefit.

In the short term, polygenic risk scores face limitations, being reliant on the quality of data obtained from GWAS: estimated effect sizes of tag variants are vulnerable to considerable stochastic variation and population structure biases [74]. However, as genetic databases become increasingly robust and include a broader range of populations, the predictive power of PRS will increase. This is likely to be further bolstered by the evolution of PRS focussed on specific pathways known to be associated to disease (e.g. the IL-23/IL-17 or antigen processing pathways). One such pathway analysis tool has recently been developed as part of the PRSice software package [75], though the concept has not yet been proven through published data. This more granular approach may offer further predictive value in disease severity and may even prove informative to clinical decision making in the choice of therapeutics.

To facilitate accreditation for implementation in the clinical laboratory, PRS will require a standardised approach applied to their methodology. PRS have poor predictive value in the context of population screening [76] and are therefore unlikely to become utilised as stand-alone tests. However, given that their positive predictive value already exceeds that of HLA-B27 testing in isolation and that testing costs are low (chip-seq analysis currently costs around $40), it appears highly probable they will fill a role in the evolving diagnostic criteria for axSpA in the near term.

## Advances in AS therapies

The use of targeted biological therapies has become the mainstay for treatment of patients with AS, who may be refractory to non-steroidal anti-inflammatory drug (NSAID) treatment. The targeting of tumour necrosis factor alpha (TNF-α), which is constitutively raised in AS patients [30, 31], is the longest established such therapy, and most widely successful. TNF-α inhibition reduces neutrophil and macrophage infiltration into the synovial fluid, arresting the inflammatory process [77, 78]. However, approximately 40% of patients either fail to respond to, or become intolerant of anti-TNF therapies [79]. As such, there is demand to develop novel targeted therapies for this group of patients.

The discovery of disease associated biomolecular pathways through the study of genetics offers an avenue for pharmaceutical exploitation. Recently, interest has surged in the application of IL-23/IL-17 pathway inhibitors in the context of AS, given the combined genetic and phenotypic evidence of this pathway’s involvement as described above [5, 80, 81]. Trials of IL-23/IL-17 pathway inhibitors to date seem to indicate that IL-17 signalling specifically is more important than IL-23 in AS [82–84]. Secukinumab (anti-IL17a) has proven effective in 70% of AS patients [82, 84], while ustekinumab (anti IL-23 and anti IL-12b) has been less useful in AS although much more promising in psoriatic arthritis (PsA). Another IL-23 inhibitor, risankizumab, has proven to be better than adalimumab (anti-TNF-α) in the treatment of psoriasis but ineffective in treating AS [83, 85]. The failure of IL-23 inhibition was an unexpected finding in the treatment of AS, given its direct upstream relationship to IL-17 and broad success in psoriasis and Crohn’s disease. The reason for this is still a subject of hot debate with no clear probable cause yet established. One possibility is that IL-17 production is being driven by alternative cytokines such as IL-1 and IL-6 [86] or that an as-yet undiscovered IL-23 independent pathway specific to entheseal tissue is driving IL-17 production. It is also possible that IL-17 inducing mechanisms may vary by tissue or disease stage [87]. While IL-17 inhibition has generally proven valuable, a proportion of patients who do not respond to TNF antagonists also fail to achieve adequate response with IL-17 inhibition [88]. Disease in these patients may be less dependent on the IL-17 pathway rendering this therapeutic approach ineffective.

Future promising novel therapeutic possibilities include Janus Kinases inhibitors (JAKinibs) which interrupt the JAK/STAT transduction of IL-23/IL-17 and other cytokine signals. A broad spectrum JAKinib, tofacitinib, has been approved for the treatment of rheumatoid arthritis, psoriasis, ulcerative colitis and more recently AS in patients with poor response to TNFi [89, 90]. Phase II & III trials for tofacitinib in AS suggest the molecule attenuates zygapophyseal inflammation on MRI, a key indicator for disease progression [89, 91]. Several other JAKinibs that could potentially be repurposed include ruxolitinib and baricitinib, which have recently been approved for the treatment of primary myelofibrosis and essential thrombocythaemia. However, due to concerns regarding the safety profile of broad spectrum JAKinibs [92], more specific JAKinibs are currently in development, including molecules specifically targeting JAK1 and TYK2. Orally administered non-biological JAK-1 kinase inhibition has shown some promise during an AS phase 2 trial [93]. TYK2 is a particularly important molecule as one of its functions is to facilitate IL-23 signal transduction. A number of compounds targeting TYK2 are currently under investigation at experimental level. Given the poor performance of IL-23 inhibition in AS so far, it is curious that human clinical trials of TYK2 inhibition to date have been shown to be effective in AS [35, 94]. Early indications suggest this specific targeting of TYK2 may also reduce side effects caused by off-target inhibition by broad spectrum JAKinibs [95, 96]. Overall, the targeting of downstream signal transduction molecules belonging to the IL-23/IL-17 axis by small inhibitory molecules offers new hope for orally administered biologic agents becoming available within several years.

## Discussion

The treatment of AS has undergone substantial change over the past two decades, in unison with our understanding of the disease and the emergence of targeted therapies. Genetics has contributed significantly to this development by revealing the molecular pathways key to the pathogenic process. Despite this, a significant proportion of patients remain difficult to treat. Genetic studies support the view that SpAs, and AS specifically, are heterogeneous diseases at the molecular level. This is exemplified by variable responsiveness to therapeutics such as those targeting IL-23/IL-17 signalling, and other consistently observed differences in clinical course including progression and severity both within and between SpA’s subtypes. Thus genetic analysis with clinical application using, for example, tailored PRS as biomarkers may be a development we will see in the short term. An obvious application would allow patients to be stratified by IL-23/IL-17 genetic risk burden to determine whether therapeutics targeting this pathway are likely to be effective.

While the genomics era has led to rapid advances in our molecular understanding of AS, significant questions remain. Much genetic evidence points squarely toward *T*-cell function; however, many more variants have yet to be functionally characterised. Further study is also required to unravel the cell types and specific biological conditions in which associated genetic variants are active. Finally, the complex web of molecular pathways and their relationship to genetic variants within these cell types and specific conditions will need to be unravelled to gain an improved understanding of the pathological process. Novel techniques such as chromatin interaction and accessibility studies, single cell gene expression and single cell proteomics may help to answer some of these questions.

Genetic associations have proven highly effective at identifying molecular pathways which are involved in the disease, but little is known about environmental exposures involved in promoting the disease or influencing clinical presentation. The close relationship between AS and IBD, together with the presence of subclinical lesions in the gut of many AS patients hint toward possible intestinal involvement. These findings have sparked considerable research into the role of gut microbiota in the disease, which has become an area of intense scientific interest.

### Limitations of this review

While this review covers a significant portion of work surrounding genetics in AS, there are further interesting avenues emerging which we have not covered here. The close relationship between AS and IBD, together with the presence of subclinical lesions in the gut of many AS patients hint toward toward the involvement of the gut as a driver of disease [97]. These findings have sparked considerable research into the role of gut microbiota in the disease, which has become an area of intense scientific interest. We have not discussed in great detail the role of innate immunity in disease aetiology. There is interesting evidence for a role of CD163 expressing M2 macrophages which may be drivers in promoting the Th17 phenotype [98]. We have also not covered the important role of the joint local metabolic microenvironment which is also thought to be important in directing lymphocyte cellular plasticity toward a pathological phenotype [99].
